# EEG-Based Emotion Classification Using Improved Cross-Connected Convolutional Neural Network

**DOI:** 10.3390/brainsci12080977

**Published:** 2022-07-24

**Authors:** Jinxiao Dai, Xugang Xi, Ge Li, Ting Wang

**Affiliations:** 1 HDU-ITMO Joint Institute, Hangzhou Dianzi University, Hangzhou 310018, China; jxdai@hdu.edu.cn; 2 Key Laboratory of Brain Machine Collaborative Intelligence of Zhejiang Province, Hangzhou 310018, China; tingwang@hdu.edu.cn; 3 School of Automation, Hangzhou Dianzi University, Hangzhou 310018, China; 4 Hangzhou Mingzhou Naokang Rehabilitation Hospital, Hangzhou 311215, China; dami20221013@163.com

**Keywords:** convolutional neural network, deep learning, electroencephalography, emotion classification, pattern identification

## Abstract

The use of electroencephalography to recognize human emotions is a key technology for advancing human–computer interactions. This study proposes an improved deep convolutional neural network model for emotion classification using a non-end-to-end training method that combines bottom-, middle-, and top-layer convolution features. Four sets of experiments using 4500 samples were conducted to verify model performance. Simultaneously, feature visualization technology was used to extract the three-layer features obtained by the model, and a scatterplot analysis was performed. The proposed model achieved a very high accuracy of 93.7%, and the extracted features exhibited the best separability among the tested models. We found that adding redundant layers did not improve model performance, and removing the data of specific channels did not significantly reduce the classification effect of the model. These results indicate that the proposed model allows for emotion recognition with a higher accuracy and speed than the previously reported models. We believe that our approach can be implemented in various applications that require the quick and accurate identification of human emotions.

## 1. Introduction

Emotion recognition has become an increasingly significant research area in the field of artificial intelligence [1,2,3]. Emotion recognition is primarily the recognition of facial expressions, speech, physiological patterns, text, and physiological signals. In this context, electroencephalography (EEG) signals, which are physiological signals, are appropriate for emotion recognition [4]. Regarding emotion classification, it was reported that the classification effect depended on the quality of the extracted features when using machine learning classification methods based on traditional features [5]. EEG has been widely used in research involving neural engineering, neuroscience, and biomedical engineering (e.g., brain–computer interfaces, sleep analysis, and disease prediction) because of its high temporal resolution, non-invasiveness, and relatively low cost [6,7]. However, the representative features of EEG signals are difficult to determine owing to their dynamic character and inter-individual differences [8].

A major problem in emotion recognition is the classification of EEG signals, which requires the extraction of appropriate features. Thus far, different approaches, such as support vector machines (SVMs) [9], general neural networks, and hidden Markov models have been applied to the classification of EEG signals [6,7]. Most of these traditional machine learning methods require considerable prior knowledge to determine the features of EEG signals. At the same time, EEG signals are vulnerable to noise interference, and EEG signals corresponding to specific behaviors may be mixed with those of other simultaneous behaviors. Particularly, in complex high-level cognitive processes, the EEG signals of individuals substantially vary, making the estimation of the representative effective features difficult in such cases. Therefore, it is extremely difficult to accurately classify EEG signals using traditional methods.

Deep learning methods have been widely used in recent years because of their ability to directly extract features in a step-by-step manner from complex data, without the need for any prior knowledge or manual feature extraction [10]. Deep learning has been applied effectively in different fields, such as image classification [11] and speech recognition [12]. The inputs for training deep networks typically fall into three categories: calculated features, images, and signal values. Feature input to EEG is often analyzed in the time–frequency domain [13]. The powers of high-alpha, high-beta, and low-beta bands, as well as low-alpha and theta waves, were shown to be significant biomarkers [14,15,16,17]. Many convolutional neural networks (CNNs) use spectrograms generated from EEG data as inputs. When signal values are used as inputs, neural networks are expected to automatically learn complex features from large amounts of data. Some researchers have applied deep learning models to EEG classification and obtained acceptable results [18,19]. Hosseini et al. [20] developed and extended a CNN structure based on principal component analysis, independent component analysis, and the differential search algorithm. They reduced the number of calculations in a baseline epilepsy dataset using this structure to extract and classify unsupervised features of big data. Meanwhile, Lan et al. [20] used a CNN to extract the features of neurological signals and classify EEG data for the resting state under open- and closed-eye conditions. Their results showed that an EEG-based biometric recognition system using a CNN can achieve high accuracy for a 10-level classification (88%). Rajendra et al. [21] employed a 13-layer deep CNN algorithm to detect the normal, preictal, and seizure classes using EEG signals. Their proposed technique exhibited an accuracy, specificity, and sensitivity of 88.67%, 90.00%, and 95.00%, respectively. Nihal et al. [22] proposed a model combining an Elman recurrent neural network (RNN) and Lyapunov exponents. Their model was used to classify the EEG signals of normal and epileptic patients, and nonlinear dynamic tools were used to calculate the Lyapunov exponent. Overall, these methods showed good classification power. On this basis, we proposed a new model and investigated the impact of high-dimensional samples and the number of layers on the performance of the model.

In this paper, we propose an improved cross-connected (C-c) CNN structural model to address the problem of using EEG signals for sentiment classification and explore the factors that affect the model performance. The innovation of this model was that three parallel structures, V1, V2, and V3, were used to extract the bottom-, middle-, and high-level features of the EEG signal, respectively, to improve the classification accuracy and speed. We conducted four experiments to assess the performance of the model: (1) We determined and compared the classification accuracies of the C-c CNN, RNN, ordinary CNN, 13-layer CNN, and long short-term memory (LSTM) models. (2) The method of feature acquisition was described, and a scatterplot of the feature separation was constructed. (3) The effects of the number of layers and the channel selection on the model performance were determined. (4) The impact of high-level samples on the model was verified. The experimental results showed that our proposed C-c CNN model exhibited a substantially better classification accuracy rate and training speed than traditional deep learning methods. We also found that the model structure of the three convolutional layers and the appropriate reduction/removal of unrelated channels increased model accuracy.

## 2. Materials and Methods

**Figure 1:**
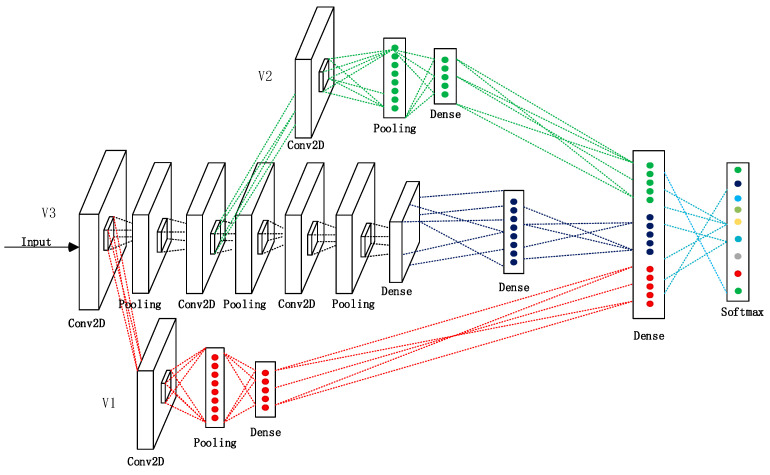
C-c CNN structure.

**Figure 2:**
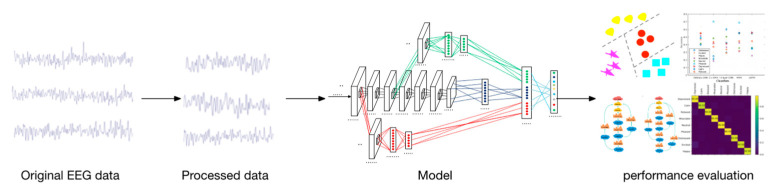
Schematic of the experimental process.

Based on the complete CNN structure [23,24,25], we constructed three independent models (V1, V2, and V3), as illustrated in Figure 1. Here, V3 is an ordinary non-C-c CNN for extracting high-level features. The first layer of the V1 and V2 sub-models was the convolutional layer, the second was the pooling layer, and the third was the fully connected layer. The sub-models V1 and V2 were separately used to extract the bottom- and middle-layer features, respectively. Subsequently, the features of the fully connected layer outputs of V1, V2, and V3 were merged into an independent feature and inputted into the softmax layer for classification. The prediction result was compared with the actual label, and the error in the loss function was calculated. Subsequently, the model was updated using the backpropagation algorithm. The experimental process is illustrated in Figure 2. The preprocessed EEG signal was inputted into the model, and the parameters were adjusted to achieve the best accuracy. Four additional experiments were conducted to verify the performance of the model.

Each EEG sample in the dataset had n channels, represented as {$x_{1},x_{2},x_{3},\ldots x_{n}$}, and each channel contained 1×m dimensional data. There were k samples and labels, denoted as {$p_{1},p_{2},\ldots ,p_{k}$}. After each training dataset was inputted into V3, the feature map $F_{1}$ was extracted using the first convolution layer w. Layer w contained n convolution kernels represented as $\left\{w_{1},w_{2},w_{3},\ldots w_{n}\right\}$. Each convolution kernel had a size of 1×3 pixels. The training of the three networks was carried out in parallel, and the bottom, middle, and top layers of the EEG signal were simultaneously extracted through V1, V2, and V3. The formula for the acquisition of $F_{1}$ can be expressed using Equation (1):

(1) $$F_{1}=\overset{n}{\underset{i=1}{\sum }}x_{i}\overset{˘}{*}w_{i}+b$$

where b denotes the bias. Next, $F_{1}$ was fed into V1 to reduce dimensionality and was thus considered as the bottom feature. Simultaneously, F1 continued to propagate in V3, and after being subsampled by the 1×2 dimensional pooling core in the second layer, the output was a 1×1×(m−4) dimensional feature map. In the pooling process, $F_{1}$ was divided into non-overlapping blocks of the size p×q. The formula for the acquisition of the (i,j)th block is expressed in Equation (2):

(2) $$maxdown\left(G_{p\times q}^{F_{1}}\left(i,j\right)\right)=max\left(a_{st}\right)$$

where $a_{st}$ denotes the value of the (s,t)th element in each convolutional region, and (i−1)·p+1≤s≤i·p, (j−1)·q+1≤t≤j·q. After passing through the third convolutional layer, the pooling feature formed a feature map $F_{2}$ with the dimensions of 1×1×(m−6). As the input of V3, $F_{2}$ underwent the same operations as in V1 to form middle-level features. Subsequently, after passing through the fourth pooling layer and fifth convolutional layer in V3, the output of V2 was a feature map $F_{3}$ with the dimensions of 1×1×(m−10), which was the top-layer feature. Finally, $F_{1}$, $F_{2}$, and $F_{3}$ were fused into a high-dimensional composite feature by the last fully connected layer of V3.

The details of the three parallel training channels of the model are presented in Table 1, Table 2 and Table 3. The Adam optimizer, configured with a learning rate of α=0.0001, was used to learn the weights. The loss function selected the categorical cross-entropy; the evaluation criterion was accuracy, the batch size was 64, and the number of epochs was 500.

The process of the algorithm is presented as below:

Input: EEG signal after being filtered and de-noised. Output: Features of bottom, middle, and top layers. The bottom-, middle-, and top-layer features of the neural network were extracted: D=40; For l in range (0,2):

(3) $$h_{l}=\sum_{i=1}^{D}x_{i}\overset{˘}{*}w_{i}+b,$$

The three-layer features were pooled and compressed through flattened and fully connected layers:

(4) $$\begin{array}{c} \lambda =1,\tau =2;\\ p=1,q=2;\\ \mathrm{For}A\mathrm{in}\mathrm{range}\;(0,2):\\ \;\;\mathrm{For}i\mathrm{in}\mathrm{range}\;(-\mathrm{int}(\mathrm{step}/2)),\;\mathrm{int}((\mathrm{step}/2)+1):\\ \;\;\;\;\;\mathrm{For}j\mathrm{in}\mathrm{range}\;(-\mathrm{int}(\mathrm{step}/2),\;\mathrm{int}(\mathrm{step}/2)+1):\\ maxdown\left(G_{p\times q}^{A}\left(i,j\right)\right)=\max\left(a_{st}\right) \end{array}$$

Three-layer features, $w_{1}$,$w_{2}$,$w_{3}$, were compressed through the fully connected layer. For n in range (0,2):

(5) $$\mathrm{Feature}=\mathrm{np}.\mathrm{hstack}(w_{n}),$$

## 3. Results

### 3.1. Dataset Description

The DEAP dataset is a large-scale EEG database jointly funded by the European Community’s Seventh Framework Program, Dutch Ministry of Economic Affairs, and Swiss National Scientific Research Foundation. It is a multimodal dataset used for analyzing human emotional states that contains the EEG data recorded for 32 participants (16 men and 16 women, with an average age of 26.9 years), watching 40 one-minute music videos showcasing different emotions. Before starting to watch, a two-minute EEG signal was collected for each subject when they were relaxed and watched the gaze cross on the screen. The sampling frequency of the EEG signal was 512 Hz, and the signals at 32 electrode positions were recorded (i.e., Fp1, AF3, F3, F7, FC5, FC1, C3, T7, CP5, CP1, P3, P7, PO3, O1, Oz, Pz, Fp2, AF4, Fz, F4, F8, FC6, FC2, Cz, C4, T8, CP6, CP2, P4, P8, PO4, and O2).

At present, there are several discrete emotion classification models, such as the six-basic-emotion-type model proposed by Ekman and Friesen [26]. Emotional dimension scales, such as the emotion wheel proposed by Plutchik [27] and Russell’s value arousal scale [28], have also been proposed. Russell’s value arousal scale was used in the abovementioned dataset. In this model, each emotional state is located on a two-dimensional plane with arousal and valence states represented along the horizontal and vertical axes, respectively. Although arousal and valence states explain most of the changes in emotional states, a third dimension of dominance can also be included in the model [29]. Arousal states can range from inactive (e.g., uninterested, bored, etc.) to active (e.g., alert, excited, etc.), whereas valence states can range from unhappy (e.g., sad, nervous, etc.) to happy (e.g., happy, elated, etc.). Dominance states range from feelings of helplessness and weakness (no control) to feelings of power (control over everything). The popular self-assessment manikin (SAM) [30] was used for self-assessment.

**Figure 3:**
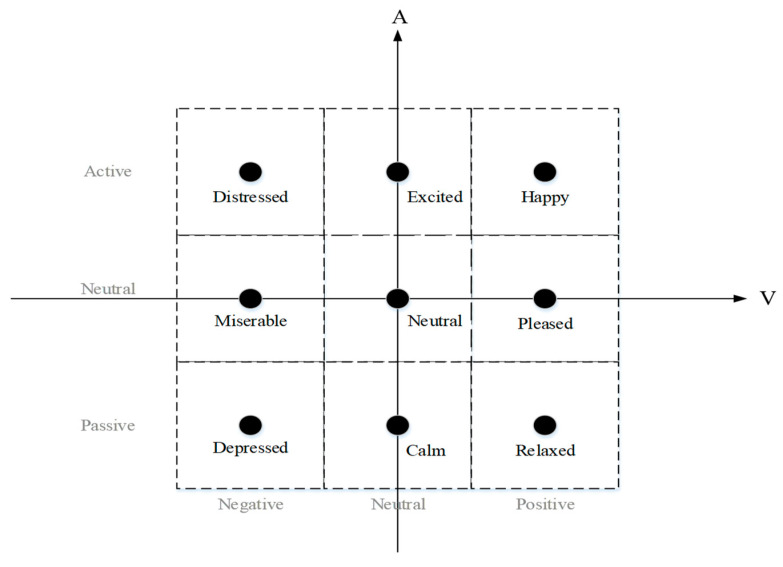
Nine emotions in the SAM scale.

In this study, a scale (ranging from 1 to 9) was mapped on three energy levels for each valence and arousal state. The valence-state values of 1–3 were mapped as “negative”, 4–6 as “neutral”, and 7–9 as “positive”. Similarly, the 1–3 arousal-scale values were mapped as “passive”, and 4–6 and 7–9 as “neutral” and “active”, respectively. According to the new proportional mapping, the model provided an emotional classification of nine states, as shown in Figure 3. The 4500 samples were evenly distributed in nine categories of emotions: depressed, calm, relaxed, miserable, neutral, pleased, distressed, excited, and happy, with 500 samples in each category.

### 3.2. Signal Preprocessing

The most useful EEG information was concentrated in the 0–30 Hz frequency range [30]. Therefore, we first filtered the original EEG signal with a low-pass filter (third-order Butterworth filter) to remove the noise in the high-frequency band and then used the wavelet threshold method to remove the EEG signal noise.

### 3.3. Experiment 1: Classification Performance of the C-c CNN

**Figure 4:**
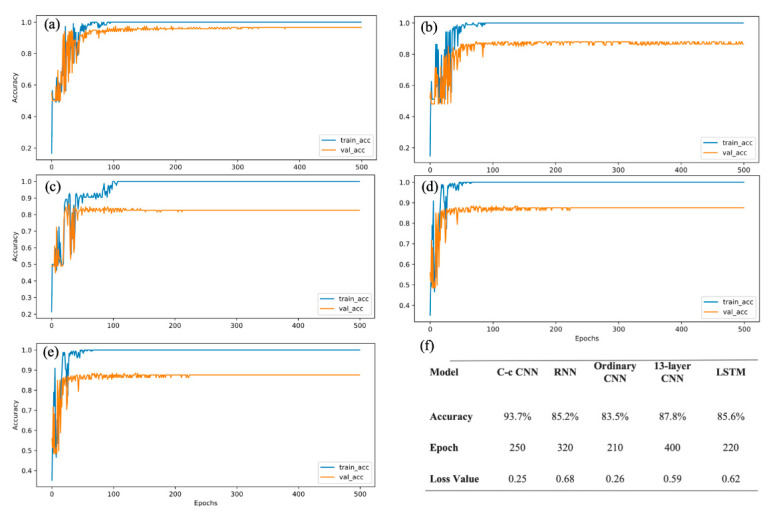
Results of Experiment 1 (**a**) C-c CNN; (**b**) RNN; (**c**) ordinary CNN; (**d**) 13-layer CNN; (**e**) LSTM; (**f**) accuracy of different classifiers.

We used several deep learning models to conduct classification experiments, including the 13-layer CNN, LSTM, RNN, C-c CNN, and non-C-c CNN (an ordinary CNN) models [21]. The experiment was conducted using Keras, with TensorFlow as the backend. The experimental results are shown in Figure 4. All experiments used tenfold cross-validation, and the training process curve was plotted for each case. Figure 4 shows that after 320 rounds of training, with the fluctuation in the RNN, the classification accuracy finally reached approximately 85.2%. For the ordinary CNN, we added a batch normalization (BN) layer and applied the dropout method. From the 210th round onward, the model exhibited a classification accuracy of 83.5% for the verification set. The accuracy of the 13-layer CNN model [21] reached 87.8% after 210 rounds; however, it showed slight fluctuations, as in the case of the RNN. The accuracy of the LSTM model was stable at 85.6% after 220 rounds of training. These experimental results showed that the convergence speed of the network was faster, and the trained results was more stable when a BN layer was used. Moreover, the number of iterations was reduced from 320 in the RNN to 210 in the proposed model, which indicated that the training time was substantially shortened. The BN layer and dropout method were used in the model presented in this study. The values of the three evaluation indicators were calculated, and the results are presented in Figure 4f.

From Experiment 1, we can conclude that the classification accuracy of the C-c CNN was substantially higher than those of the currently popular deep learning models or the traditional CNNs. The addition of the C-c convolutional layer merged the feature information of different layers and improved the classification performance of the model. In this regard, Sohaib [31] used only the sample data for five participants and trained a classifier model to obtain a classification accuracy of 77.78%. Compared with the two-category classification CNN in [32], our C-c CNN model demonstrated all nine classifications with a substantially improved accuracy rate.

### 3.4. Experiment 2: Use of Non-End-to-End Methods to Obtain Different Levels of Features

**Figure 5:**
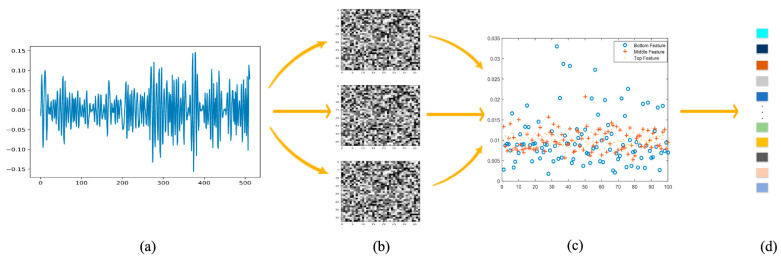
Feature extraction schematic: (**a**) original signal; (**b**) convolution kernel; (**c**) distribution scatterplot of the three-layer features; (**d**) new high-dimensional feature.

In the second experimental phase, we used a Python toolkit to determine the shape of the convolutional core of the network, as shown in Figure 5. The first, second, third, and fourth columns show the original signal map of the input data, shape of the convolutional kernel after training, distribution scatterplot of the three-layer features, and new high-dimensional features after fusion, respectively. The input data were signals with dimensions of 40×8064. After the feature extraction of the three parallel layer channels, the luminance arrangement of the convolution kernel was gradually abstracted, the shape of the convolution kernel in the lower layer was regular, and the bright spot distribution of the convolution kernel at the high level became chaotic. This result showed that the convolution kernel was significantly affected by the details of the abstract component of the input data and extracts its features. Unlike the method reported by Samarth [32], which transformed the input signal into a two-dimensional image and performed feature extraction with 3×3 convolution kernels, we directly inputted the one-dimensional EEG signal and applied a convolution kernel with the dimensions of 1×3 for feature extraction. After passing through 40×1×3 convolution kernels and pooling kernels, the input EEG data were transformed into a feature map and then compressed into a 1×100 output by the fully connected layer. Finally, the bottom, middle, and top layer features were combined into a comprehensive feature with the dimensions of 1×300.

As mentioned above, Figure 5c shows the bottom-, middle-, and top-layer features of a sample in the form of a scatter diagram. The bottom-layer features of the data extracted from the first channel were widely distributed. The middle-layer feature extracted by the second channel was more “compact” than the bottom-layer feature distribution, and the upper-layer feature was even more closely distributed. The features extracted by the CNN were increasingly concentrated in the region of interest from the lower to higher levels; however, some features were ignored during abstraction. Therefore, a C-c CNN was used to synthetically consider the features of the low, middle, and high levels to achieve better classification.

**Figure 6:**
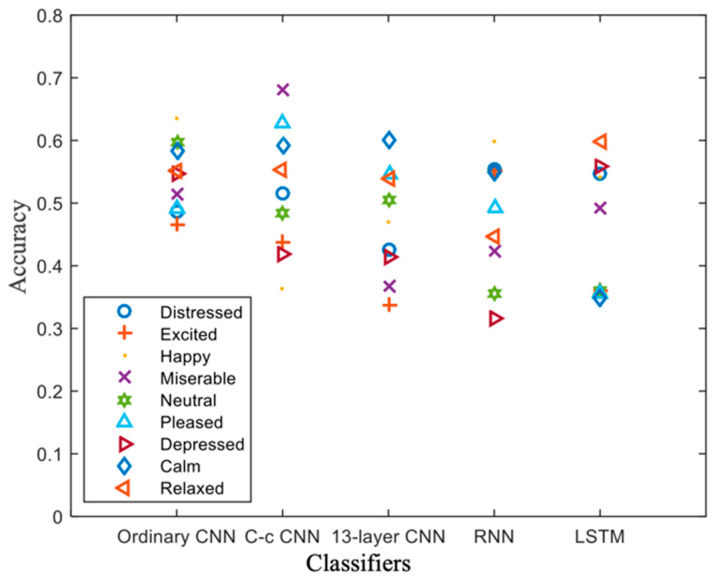
Feature separation scatter diagram.

Next, we extracted the features and obtained feature scatterplots for the EEG signals of nine different emotions (Figure 6). The features extracted from our model exhibited better separability than those of the other models.

### 3.5. Experiment 3: Effect of the Number of Layers on Model Performance

**Figure 7:**
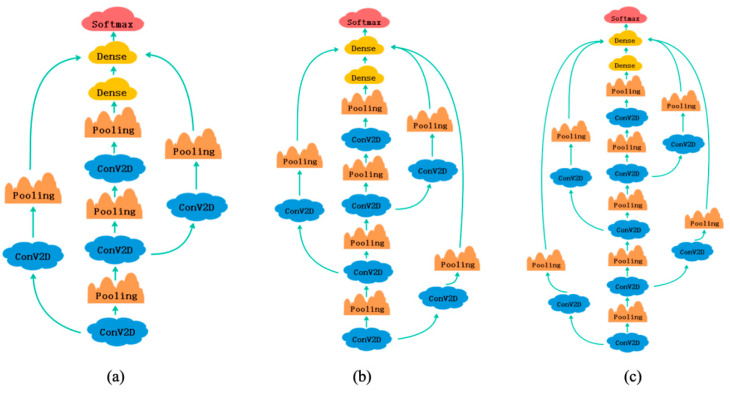
Experiments using C-c CNN models with different numbers of layers: (**a**) basic C-c CNN, (**b**) C-c CNN with one added layer, and (**c**) C-c CNN with two added layers.

In the third experimental phase of the study, we considered three different depth models that were derived by adding none, one, and two layers of channels to the C-c CNN, as shown in Figure 7.

The powerful feature extraction ability of deep learning is largely explained by the large number of layers used in the model. However, in our case, we found that adding more layers to the cross-linked CNN and extracting more levels of features did not improve classification accuracy, thus making the newly added layers functionally redundant.

**Figure 8:**
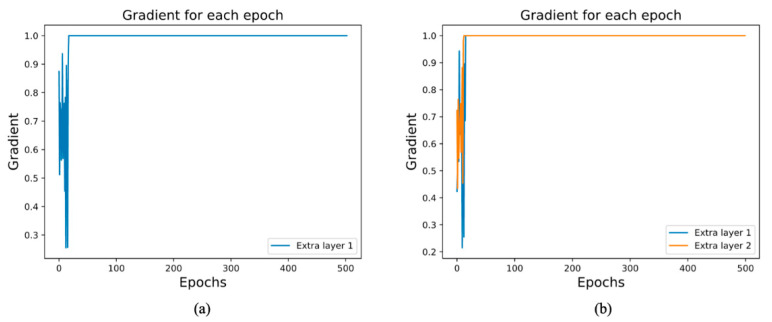
Change in gradient with (**a**) one redundant layer and (**b**) two redundant layers.

Subsequently, we extracted the gradient of the excess layer, as shown in Figure 8. From the line graph, we noted that when the extra layer was backpropagated to update the weights, the layer gradient was maintained at 1, which meant that the layer weight was not updated during training. The three-layered C-c CNN extracted all features of interest. The new test layers were completely redundant and did not aid in model classification; the new layer decreased the model performance. This situation arose not because of overfitting, but because of the same problem as that underlying the ResNet reaction. Thus, it is not always better to have more layers, as the structure of the three convolutional layers was sufficient to extract the required features.

The experimental results showed that the extra layers were equivalent to identity mapping. During forward propagation, the initialization weights were obtained. However, when the parameters were updated backwards, they remained unchanged after several parameter updates, until the model training was completed.

### 3.6. Experiment 4: Effect of High-Dimensional Samples on the Model

In the final experimental phase of the study, we examined the effects of dataset dimensions on the performance of the proposed model. The DEAP dataset used a 32-channel BioSemi activation device to collect EEG signals from the subjects. Recent studies have shown that subjective positive emotions are closely related to the prefrontal and anterior cingulate cortexes, and that negative emotions involve whole-brain systems, with each emotion dependent upon specific nervous systems and brain regions [33,34,35]. At this stage, we investigated the effect of the number of EEG channels on model classification by providing the original dataset and a dataset with several channels removed from the neural network. The specific method was to compare the original data, retaining only the frontal lobe data (the removal of data corresponding to the P3, P4, PZ, CPZ, CP3, and CP4 channels) and retaining only the occipital lobe data (the removal of data corresponding to the F3, F4, FZ, FP1, FP2, and FCZ channels).

**Figure 9:**
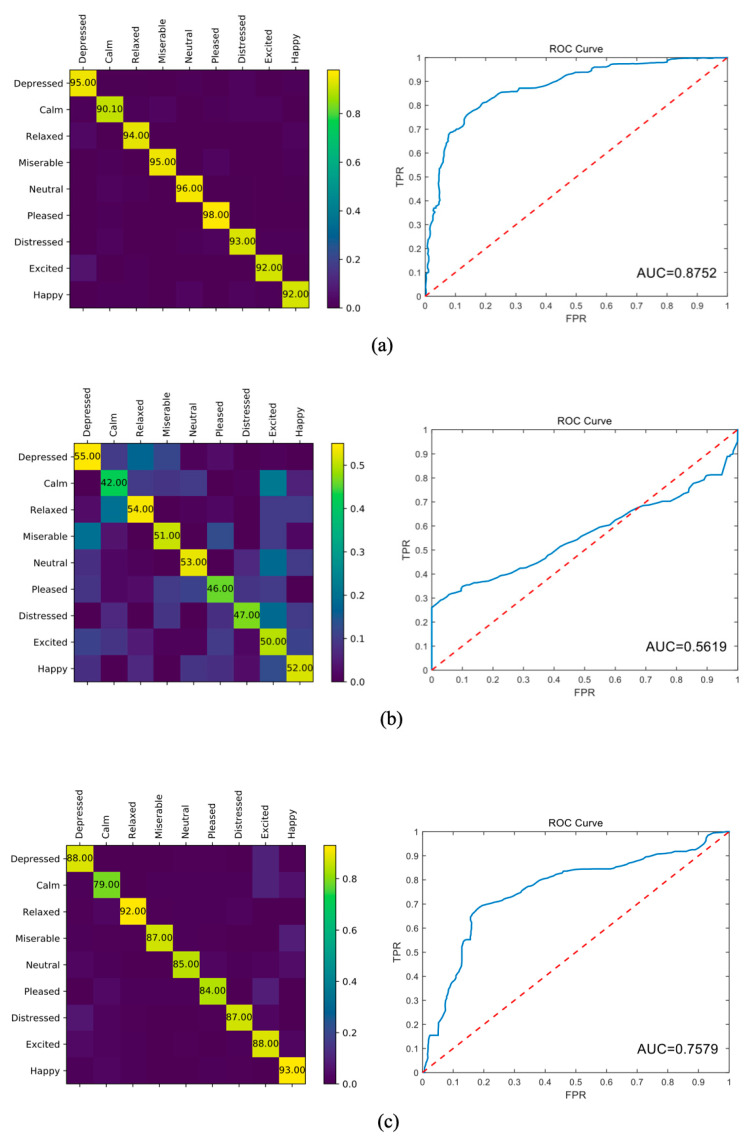
Results of the experiments with different dimensional datasets. Results obtained with (**a**) raw data; (**b**) removal of data corresponding to channels P3, P4, PZ, CPZ, CP3, and CP4; (**c**) removal of data corresponding to channels F3, F4, FZ, FP1, FP2, and FCZ.

For this experiment, we plotted the confusion matrix and receiver operating characteristic (ROC) curve for the analysis, as shown in Figure 9. Because emotion classification is a multiclassification problem, the method of drawing an ROC curve is different from that of the two-class problem. First, we preprocessed all labels using one-hot encoding. The preprocessing labels consisted of only zero and one, where the position of one indicated its category (corresponding to “positive” in the two-category problem) and zero indicated other categories (corresponding to “negative” in the two-category problem). If the classifier classified the test sample correctly, the value of the position corresponding to one in the sample label in the probability matrix was greater than that corresponding to zero.

Based on the two aforementioned points, the label and probability matrices were expanded in rows, and two columns were generated after transposition, corresponding to the results of the two classifications. Therefore, this method was used to directly obtain the final ROC curve after calculation.

We studied the effects of three EEG channel distributions. As shown in Figure 9, the removal of data on channels F3, F4, FZ, FP1, FP2, and FCZ from training produced almost no impact on the model; only the convergence speed increased, with a slight reduction in accuracy. However, removing the data on channels P3, P4, PZ, CPZ, CP3, and CP4 drastically reduced the model performance.

**Figure 10:**
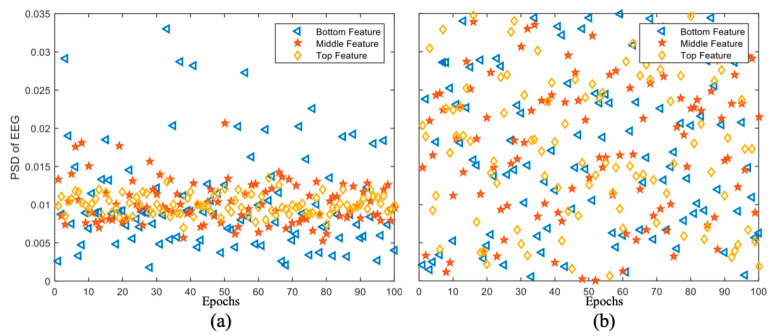
Feature distribution map with (**a**) the removal of data from channels P3, P4, PZ, CPZ, CP3, and CP4; (**b**) removal of data removed from F3, F4, FZ, FP1, FP2, and FCZ.

We speculate that this is because the features extracted by the different channels were different in the removed channels, and only a few specific channels may have contained important information. Thus, we separately extracted the data from different channels and inputted them into our model for analysis. We extracted the feature distribution maps from the data in Figure 9b,c, as shown in Figure 10, and calculated the power spectral density (PSD) features of the EEG signals.

The feature distribution in Figure 10a is similar to that of the original dataset. By contrast, in Figure 10b, because the channels containing important information were removed, the extracted feature distribution became chaotic, and the information of different scales was mixed, which significantly affected the model performance.

From the confusion matrix, we noted that the classification accuracy of the model significantly decreased when data from the P3, P4, PZ, CPZ, CP3, and CP4 channels were removed. The labels were tagged incorrectly. However, when the F3, F4, FZ, FP1, FP2, and FCZ channel data were removed, the model was only unable to correctly classify a small number of “Pleased”, “Excited”, and “Happy” tags, or “Relaxed” and “Calm” tags. We speculate that this result may be related to the regional division of brain function. The P3, P4, PZ, CPZ, CP3, and CP4 channels are distributed near the thalamus, which controls emotional expression, while the F3, F4, FZ, FP1, FP2, and FCZ channels are located in the forehead, far away from the area controlling emotion [33,35]. The removal of channels in the “emotion region” resulted in a significant loss of information, which reduced classification accuracy.

**Figure 11:**
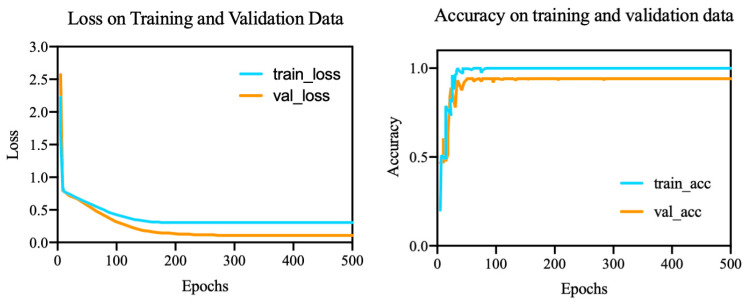
Loss and accuracy.

**Figure 12:**
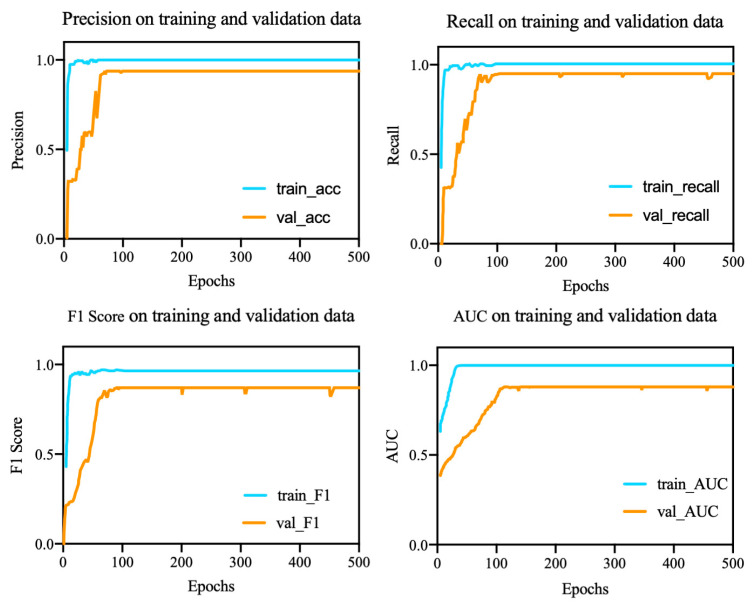
Model performance evaluation.

Figure 11 shows the loss function and the accuracy of the model. As the epoch increased, the loss function gradually decreased and reached a steady-state value after the 180th epoch. The accuracy tended to stabilize as the epochs approached 100. We used ten-fold cross-validation. The precision, F1 score, recall, and area under the ROC curve (AUC) were used as evaluation criteria for the model, and the results are shown in Figure 12.

As shown in Table 4, the average accuracy of the proposed model was 93.7%, the overall standard deviation was 0.171, and the precision, recall, F1 score, and AUC were 89.6%, 88.1%, 88.8%, and 91.9%, respectively.

## 4. Discussion and Conclusions

In general, although the extraction of features in traditional learning methods has good interpretability, it is cumbersome, requires professional expertise, and may still result in the incomplete detection of features. Deep learning can automatically extract features through model training and has strong robustness, adaptability, and comprehensive information-processing capabilities.

In this study, we proposed an improved C-c CNN model to address the problem of using EEG signals for emotion classification and explored the factors affecting model performance. Traditional artificial feature extraction methods are too slow for application in real-time emotion classification. Compared with traditional classifiers, deep learning substantially improved classification accuracy. Moreover, there is no need to manually extract features, and deep learning can satisfy the requirements of rapid acquisition of classification results in practical applications. Our model used a cross-continuous convolution layer and a 40 × 1 × 3 convolution kernel to fuse EEG features of different scales and improve recognition performance. Compared with common classification methods, our proposed method exploited techniques, such as dropout, to achieve a higher classification accuracy with the DEAP dataset. EEG emotion recognition research based on C-c CNNs uses preprocessed EEG signals as inputs. However, the raw EEG signal cannot reflect the positional relationship between EEG channels, nor can it distinguish the effects of high-level samples on the model. Therefore, we supplemented related experiments to verify the effect of the number of layers, high-dimensional samples, and channel selection on the model.

Table 5 shows a comparison of the proposed model with the previously reported EEG-based techniques for emotion classification using the DEAP dataset. The table clearly shows that our model achieved higher accuracy than most of the previous models using the same dataset and can classify significantly more emotions.

In this study, the C-c CNN network constructed using V1, V2, and V3, extracted the features of the complex network, and the classification accuracy of nine emotions reached 93.7%.

Therefore, the premise of our experiments was that all expressed emotions are unique and identifiable. The limitations of this study were that the features of the output could not be explained and that the application of emotion recognition required us to quickly identify emotions. Although the number of training epochs required for the proposed model was significantly lower than those of traditional CNN models after using BN layers, the efficiency of running a program in a Python editor was limited, as our model needed to extract the bottom-, middle-, and top-layer features of the data three times. In the future, we plan to apply multi-GPU technology to solve the problem of low model efficiency. We also plan to use our proposed model for the online classification of emotions to obtain suitable initial network weights, which can significantly reduce the time required for training initialization weights.

## Figures and Tables

**Table 1:** Bottom-channel network structure diagram.

Layer	Type	Kernel	Stride	Output Size
I	Input			40 × 1 × 8064
L1	Conv2D	40 × 1 × 3	1	1 ×1 × 8062
L2	Pooling	1 × 2	2	1 ×1 × 4031
L3	Dense	1 × 100		1 × 100
O	Dense	1 × 9		1 × 9

**Table 2:** Middle-channel network structure diagram.

Layer	Type	Kernel	Stride	Output Size
I	Input			40 × 1 × 8064
L1	Conv2D	40 × 1 × 3	1	1 × 1 × 8062
L2	Pooling	1 × 2	1	1 × 1 × 4031
L3	Conv2D	1 × 3	1	1 × 1 × 4029
L4	Pooling	1 × 2	2	1 × 1 × 2015
L5	Dense	1 × 100		1 × 100
O	Dense	1 × 9		1 × 9

**Table 3:** Top-channel network structure diagram.

Layer	Type	Kernel	Stride	Output Size
I	Input			40 × 1 × 8064
L1	Conv2D	40 × 1 × 3	1	1 × 1 × 8062
L2	Pooling	1 × 2	1	1 × 1 × 4031
L3	Conv2D	1 × 3	1	1 × 1 × 4029
L4	Pooling	1 × 2	1	1 × 1 × 2015
L5	Conv2D	1 × 3	1	1 × 1 × 2013
L6	Pooling	1 × 2	1	1 × 1 × 1007
L7	Dense	1 × 100		1 × 100
O	Dense	1 × 9		1 × 9

**Table 4:** Results of the classification performance.

Model	Accuracy	Precision	Recall	F1	AUC
C-c CNN	93.7%	89.6%	88.1%	88.8%	91.9%

**Table 5:** Classification accuracies of different approaches.

Research	Features	Method	Number of Emotion Categories	Average Accuracy (%)
Mert and Akan (2018) [36]	Time–frequency	SVM	2	82.05
Hong Zeng (2019) [37]	Time–frequency	SincNet-R	3	94.50
Hayriye Donmez(2020) [38]	Frequency	CNN	3	84,69
WenKai Huang (2020) [39]	Time–frequency	S-EEGNet	2	89.11
Yuling Luo (2020) [40]	Time–frequency	NeuCube	4	88.12
Hanjie Liu (2022) [41]	Complex network	GNN	2	92.31
This work	Complex network	C-c CNN	9	93.70

## Data Availability

Publicly available datasets were analyzed in this study. This data can be found here: [http://www.eecs.qmul.ac.uk/mmv/datasets/deap/readme.html], accessed on 20 June 2022.
